# Effects of Ibuprofen and Diclofenac Pre-Treatment on Viability and Apoptosis Processes in Human Dental Pulp Stem Cells

**DOI:** 10.3390/medicina60050787

**Published:** 2024-05-09

**Authors:** Adriana Adamičková, Jan Kyselovic, Matúš Adamička, Nikola Chomaničová, Simona Valášková, Barbara Šalingová, Miroslava Molitorisová, Zdenko Červenák, Ľuboš Danišovič, Andrea Gažová

**Affiliations:** 1 5th Department of Internal Medicine, Faculty of Medicine, Comenius University Bratislava, Špitálska 24, 81372 Bratislava, Slovakia; 2 Department of Pharmacology and Toxicology, University of Veterinary Medicine and Pharmacy, 04181 Košice, Slovakia; 3 Institute of Medical Biology, Genetics and Clinical Genetics, Faculty of Medicine, Comenius University Bratislava, Špitálska 24, 81372 Bratislava, Slovakia; matus.adamicka@gmail.com (M.A.);; 4 International Laser Centre, Slovak Centre of Scientific and Technical Information, Lamačská cesta 7315/8A, 84104 Bratislava, Slovakia; 5 Institute of Pharmacology and Clinical Pharmacology, Faculty of Medicine, Comenius University Bratislava, Špitálska 24, 81372 Bratislava, Slovakia

**Keywords:** dental pulp stem cells, nonsteroidal anti-inflammatory drugs, ibuprofen, diclofenac, angiogenesis, apoptosis

## Abstract

*Background and Objectives*: Stem cell-based regeneration strategies have shown therapeutic efficacy in various fields of regenerative medicine. These include bone healing after bone augmentation, often complicated by pain, which is managed by using nonsteroidal anti-inflammatory drugs (NSAIDs). However, information is limited about how NSAIDs affect the therapeutic potential of stem cells. *Materials and Methods*: We investigated the effects of ibuprofen and diclofenac on the characteristics, morphology, and immunophenotype of human mesenchymal stromal cells isolated from the dental pulp (*DPSCs*) and cultured in vitro, as well as their effects on the expression of angiogenic growth factors (*VEGFA* and *HGF*) and selected genes in apoptosis signalling pathways (*BAX*, *BAK*, *CASP3*, *CASP9*, and *BCL*2). *Results*: Ibuprofen and diclofenac significantly reduced the viability of DPSCs, while the expression of mesenchymal stem cell surface markers was unaffected. Both ibuprofen and diclofenac treatment significantly upregulated the expression of *HGF*, while the expression of *VEGFA* remained unchanged. Ibuprofen significantly altered the expression of several apoptosis-related genes, including the upregulation of *CASP9* and *BCL2*, with decreased *CASP3* expression. BAK, CASP3, CASP9, and BCL2 expressions were significantly increased in the diclofenac-treated DPSCs, while no difference was demonstrated in BAX expression. *Conclusions*: Our results suggest that concomitant use of the NSAIDs ibuprofen or diclofenac with stem cell therapy may negatively impact cell viability and alter the expression of apoptosis-related genes, affecting the efficacy of stem cell therapy.

## 1. Introduction

Stem cell-based tissue engineering approaches offer important therapeutic modalities in numerous medical disciplines. These include bone augmentation in oral/maxillofacial surgery for bone defect treatment and periodontal reconstruction [1,2]. The most common treatments to restore extensive bone loss and defects are autogenous bone grafts, allograft, xenograft, isograft, or alloplastic material, which remain a standard procedure for significant defects [3]. However, these approaches have many limitations and shortcomings in the morphological and functional reconstruction of defects [4,5].

To overcome the difficulties associated with grafting procedures, intensive research and development of bone graft alternatives employ different combinations of osteoconductive materials, growth factors, and stem cells [6]. The goal of regenerative medicine is to replace the damaged area with tissue specific to the patient at cellular and immunological levels. Therefore, a promising alternative to standard therapy is using collagen scaffolds in combination with cells possessing osteogenic potential, i.e., stem cells [7,8].

Mesenchymal stem cells (MSCs) are multipotent stromal stem cells that can be harvested from various sources and differentiated into multiple cell types, such as osteogenic chondroblasts and osteoblasts [9]. The beneficial therapeutic effects of MSCs are due to their ability to support a regenerative microenvironment through immunomodulatory effects, stimulation of angiogenesis and antiapoptotic effects, as well as the recruitment of host stem/progenitor cells into the site of bone repair [10,11]. The efficacy of regenerative therapy using stem cells depends on several factors, including the delivery method, the concentration of stem cells per injection, the carrier used, and the extent of injury [12,13]. Moreover, the outcome of regenerative therapy depends on a combination of the interactions between transplanted MSCs and the recipient’s cellular and molecular components, as well as any current pharmacotherapy of the patient with effects on MSC and bone microenvironment [14].

Clinical recommendations for the postsurgical pain after bone augmentations in oral surgery include nonsteroidal anti-inflammatory drugs (NSAIDs), such as ibuprofen (IBU) and diclofenac (DIC) [15]. Despite their extensive use, a clear understanding of their mechanism is still lacking. The exact mechanism of action is not entirely known. Still, the primary mechanism responsible for anti-inflammatory, antipyretic, and analgesic action is the inhibition of prostaglandin synthesis by inhibiting cyclooxygenase (COX) [16]. Some studies suggested that NSAIDs have inhibitory effects on MSCs, specifically the secretion of regenerative factors [17,18]. MSCs dynamically respond to the microenvironment of the injury and are carried by altering their secretory profile, thereby restoring homeostasis. We investigated the effects of ibuprofen and diclofenac on the characteristics, morphology, and immunophenotype of human mesenchymal stromal cells isolated from the dental pulp (DPSCs) and cultured in vitro, as well as their effects on the expression of angiogenic growth factors (VEGFA and HGF) and selected genes in apoptosis signalling pathways (BAX, BAK, CASP3, CASP9, and BCL2).

## 2. Materials and Methods

### 2.1. Cell Isolation

Cells were isolated from the dental pulp of extracted third molars from healthy donors (N = 4, age 18–20) according to the Helsinki Declaration after the donor’s informed consent and approval by the local ethical committee of the Hospital Ruzinov, Bratislava, Slovakia. The teeth were removed due to orthodontic therapy (not because of the experiment), and no pathological alterations were observed.

Pulp tissues were washed thoroughly in PBS containing antibiotics and cut into 1–2 mm^2^ pieces. Small pieces were placed in 60 mm culture dishes in a random pattern. Drops of foetal bovine serum (FBS, Sigma Aldrich, Taufkirchen, Germany) were applied on the tissues, sufficient to cover them entirely, and maintained at 37 °C in a humidified incubator with 5% CO_2_. After 2 h incubation, explants were maintained in low-glucose DMEM (Dulbecco’s modified Eagle medium, Sigma Aldrich, Germany) enriched with 10% FBS, penicillin (100 IU/mL, Sigma Aldrich, Germany), and streptomycin (100 µg/mL, Sigma Aldrich, Germany). The culture medium was changed every 2–3 days, and the cell outgrowth was monitored regularly with an M-795 inverted microscope (OPTIKA S.R.L., Ponteranica, Italy). The outgrown cells at 70–80% confluence were detached using 0.25% Trypsin-EDTA solution and transferred to a T-75 flask. Between 4 and 6 passages were performed for each experiment.

### 2.2. hDPSCs’ Characterisation

To confirm the phenotype of isolated cells, every hDPSC population at the 3rd passage was identified by flow cytometry (MACS Quant Analyzer, Miltenyi Biotec, Bergisch Gladbach, Germany) according to the phenotypic signature described by the Mesenchymal and Tissue Stem Cell Committee of the International Society for Cellular Therapy (ISCT) using the MSC Phenotyping kit (Miltenyi Biotec, Germany) according to manufacturer’s instructions. Cell viability was assessed with propidium iodide (PI, Miltenyi Biotec, Germany). The morphology of DPSCs was evaluated with microscopic observations using the M-795 inverted microscope (OPTIKA S.R.L., Italy). For the experiment, 0.5 × 10^6^ cells of the required passage were transferred to a cell culture plate. After pharmacological treatment, DPSCs were evaluated by flow cytometry, as described above. Medians with 25–75% percentiles were calculated using two experiments with cells from different donors (N = 4).

### 2.3. Ibuprofen and Diclofenac Treatment

For all experiments presented in the study, an ibuprofen (IBU) stock [19] (Sigma Aldrich, Germany) at a concentration of 50 mg/mL (240 mM) in ethanol and a diclofenac (DIC) stock [20] (Sigma Aldrich, Germany) at a concentration of 50 mg/mL (157 mM) in methanol was used. The stock solutions were prepared freshly before each experimental setup.

According to the literature data [21,22,23,24,25], we selected concentrations of IBU of 150 µM and 300 µM and DIC of 1.5 µM and 3 µM to be administered to DPSCs for treatment at 24, 48, and 72 h.

The control groups (CTRL) in all IBU experiments were hDPSCs treated with equal amounts of solvent (ethanol) in the medium that was in the tested samples. The final solvent concentration in samples achieved 0.06% (*v*/*v*) in samples treated with IBU 150 µM and 0.125% (*v*/*v*) in samples with IBU 300 µM. The control groups in all DIC experiments were hDPSCs treated with equal amounts of solvent (methanol) in the medium that was in the tested samples. The final solvent concentration in samples achieved 0.0008% (*v*/*v*) in samples treated with DIC 1.5 µM and 0.0019% (*v*/*v*) in samples with DIC 3.0 µM.

### 2.4. Immunofluorescence Staining

DPSCs (1 × 10^4^ cells/well) were seeded on coverslips in 12-well plates and cultured as described above to evaluate morphological changes induced by different treatments. After seven days, cells were fixed with 4% paraformaldehyde and permeabilised with 0.2% Triton X/0.1% Tween/1xPBS (30 min). The cells were then blocked with 5% goat serum (Sigma Aldrich, Germany) and incubated with rabbit vimentin antibody (1:100 dilution; cat. No D21H3 XP, Cell Signalling Technology, Danvers, MA, USA). Alexa Fluor 488-conjugated Goat anti-mouse IgG (H + L) (1:500 dilution; Cell Signalling Technology, USA) was used to incubate the cells at room temperature for 2 h. DAPI (10 min) was used to stain cell nuclei. Fluorescent images were captured on a Ti-E microscope (Nikon instrument, Boston, MA, USA) at 40× magnification.

### 2.5. MTT Assay

To determine the cytotoxicity effect and proliferation rate of hDPSCs in culture after IBU and DIC treatment, the tetrazolium salt (MTT) reduction test was carried out with a cell proliferation kit (Sigma Aldrich, Germany). Cells were seeded in 96-well plates at 3 × 10^4^ cells per well in a standard culture medium. Cells were incubated for 24 h, and after the incubation period, cells were treated with IBU and DIC, as described above. All conditions were performed in quadruplicate. After incubation (24, 48, and 72 h), 10 µL of the MTT labelling reagent (final concentration 0.5 mg/mL) was added to each well. Following 4 h of incubation, 100 µL of the solubilisation solution was added to each well with a 24 h incubation period. The absorbance was measured at a wavelength of 570 nm with a Varioskan LUX microplate reader (Thermo Fisher Scientific, Waltham, MA, USA).

### 2.6. Gene Expression

Total RNA was isolated from DPSCs using the Tri-Reagent^®^ (Sigma Aldrich, Germany) with phenol-chloroform extraction and quantified using the Qubit RNA XR Assay Kit (Thermo Fisher Scientific, USA) according to the manufacturer’s instructions. Then, 3000 ng of nucleic acid was transcribed into cDNA using the High-Capacity cDNA Reverse KIT with RNAse inhibitor (Thermo Fisher Scientific, USA). MRNA expression was quantified using TaqMan Universal PCR Master Mix kit on the QuantStudio 5 Real-Time PCR system (Thermo Fisher Scientific, USA) and probes for the following genes (Table 1).

The Pfaffl method was used to calculate the relative expression [26]. Results were normalised to the geometric mean of the two most suitable reference genes B2M and GAPDH (Table 1) [27]. Calculated normalised quantities were calibrated to appropriate control groups. Medians with 25–75% percentiles were calculated based on two experiments with cells from different donors (n = 4) performed in triplicates.

### 2.7. Statistical Analysis

Statistical analysis was performed using GraphPad Prism version 5.00 (GraphPad Software, San Diego, CA, USA). Values in text are presented as mean ± SEM, all values in graphs are presented as medians with 25–75% percentiles, and statistical analysis was performed comparing treated DPSCs with appropriate controls. The Shapiro–Wilk test was used to determine data distribution. We used the Kruskal–Wallis and Dunn’s tests to compare more than two groups. Student’s *t*-test determined the statistical significance between the two groups. *p*-value ≤ 0.05 was considered statistically significant.

## 3. Results

### 3.1. Characterisation of Isolated DPSC Populations

**Figure 1:**
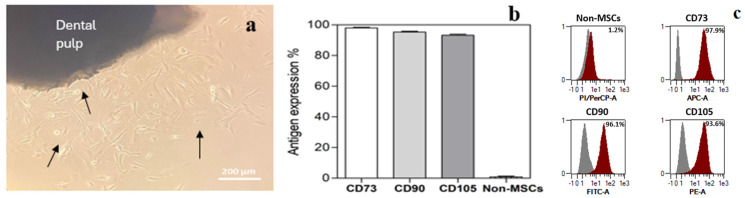
Characterisation of DPSCs. (**a**)—Primary cell colonies growing from dental pulp were observed 20 days after initial seeding (arrows marked spindle-shaped cells). (**b**)—DPSCs expressed characteristic mesenchymal stem cell markers (CD73, CD90, and CD105), while the non-MSC markers (CD14, CD20, CD34, and CD45) were not detected. (**c**)—Representative overlay histograms of DPSCs show the control populations (grey) and the specifically stained cells (red).

All cell populations isolated from human dental pulps used in the experiments displayed mesenchymal-like morphology involving spindle-shaped cell types (Figure 1a). The immunophenotype evaluated using flow cytometry showed specific surface antigen expression typical for MSCs (CD90, CD 73, and CD 105), while more than 98% of cells were negative for the non-MSC markers (CD14, CD 20, CD34, and CD45) (Figure 1b,c).

### 3.2. Effect of Ibuprofen and Diclofenac Pre-Treatment on Characteristics, Morphology, and Immunophenotype of DPSCs

**Figure 2:**
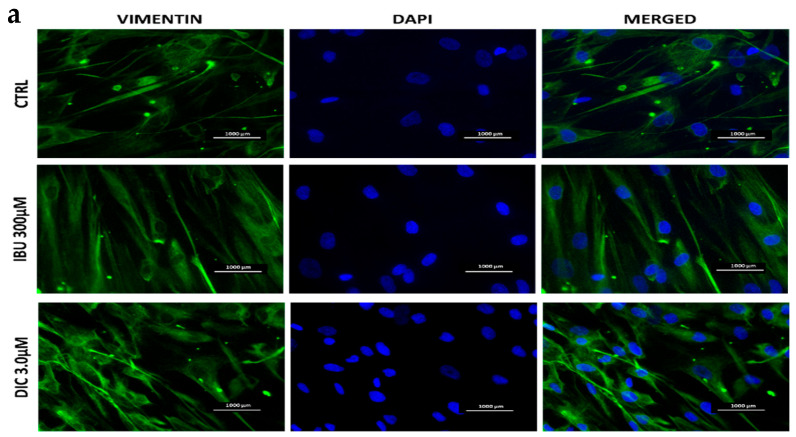

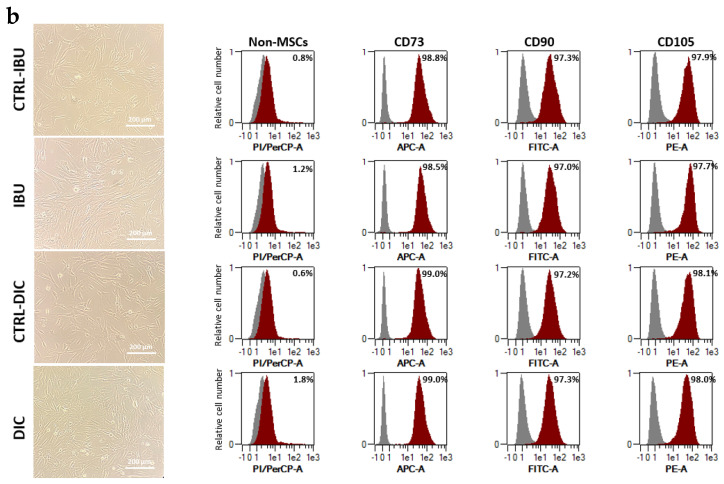
Characterisation of cultured DPSCs. (**a**)—Fluorescence microscopy of DPSCs cultured with IBU and DIC after 48 h and stained for vimentin (green). Cell nuclei were counterstained with DAPI (blue). (**b**)—Morphology of DPSCs at light microscopic levels, representative overlay histograms of DPSCs treated with IBU or DIC or CTRL populations (grey) and the specifically stained cells (red).

Immunofluorescence staining of untreated and pre-treated DPSCs was used to investigate changes in the expression of the cytoskeletal intermediate filament vimentin and nuclei of cells. Treatment with NSAIDs showed no significant differences in expression between DPSC groups (Figure 2a). Regarding cellular morphology, images showed elongated cells with no significant morphological difference between groups. Surface markers were analysed with flow cytometry, with no differences in expression observed between NSAID-treated and untreated DPSCs (Figure 2b).

### 3.3. Effect of Ibuprofen and Diclofenac Pre-Treatment on Viability of DPSCs

**Figure 3:**
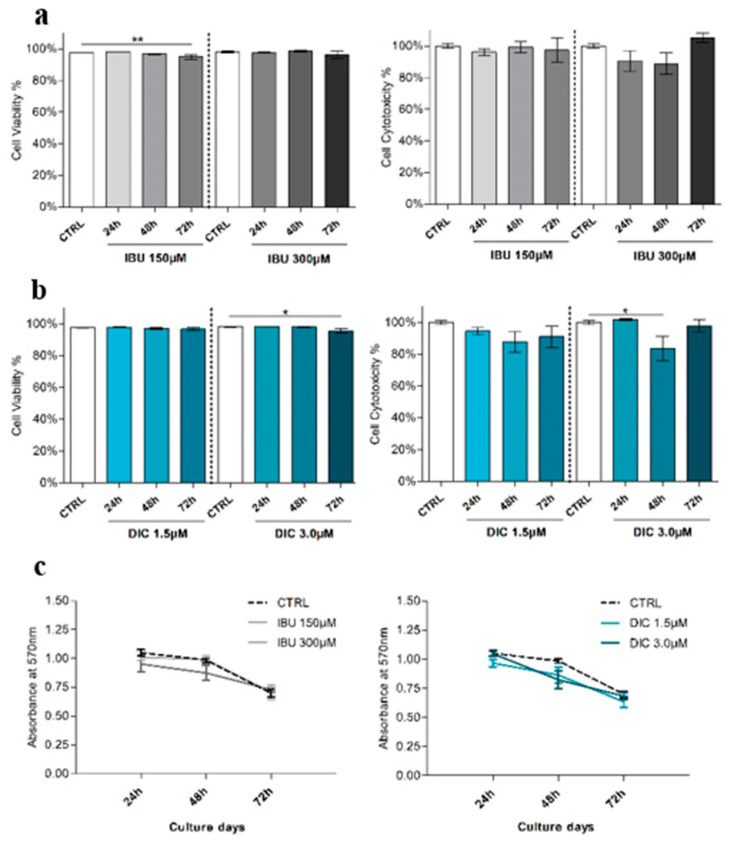
Viability and morphology of DPSCs treated with IBU and DIC. (**a**)—DPSCs treated with IBU and DIC cell viability after 24, 48, and 72 h. Cell viability was assessed by flow cytometry. (**b**)—The effect of IBU and DIC on the cytotoxicity of hDPSCs was evaluated with an MTT assay. (**c**)—The proliferation rate of hDPSCs after NSAIDs treatment was assessed by an MTT assay. Data are presented as means ± SEM (n = 4). The Mann–Whitney test was used to perform statistical analysis. *p* * ≤ 0.05 or *p* ** ≤ 0.01 compared to the CTRL group.

Viability was evaluated by flow cytometry using propidium iodide staining to determine the effect of IBU and DIC pre-treatment on DPSCs. Cell viability analysis showed decreasing patterns in both treatments of DPSCs (Figure 3a,b). DPSCs cultivated with IBU 150 µM for 72 h resulted in significantly reduced cell viability by 2.5% (mean 94.9 ± 1.4%) compared to the control (mean 97.4 ± 0.16%, *p* ≤ 0.0061). Similarly, DIC 3 µM treatment for 72 h led to a 2.58% decrease in viability (mean 95.6 ± 1.4%) compared to the CTRL (mean 98.1 ± 0.19%, *p* ≤ 0.0242) (Figure 3a,b).

The reduction in the survival of hDPSCs after IBU treatment was not observed in the cytotoxicity assay. The decrease in the viability of hDPSCs was observed in DIC 3 µM for 48 h intervals (mean 83.6 ± 7.7%) compared to the CTRL (100 ± 1.4%, *p* ≤ 0.043).

An MTT assay (Figure 3c) was performed to assess potential differences in the proliferation rate between DPSC groups. The results revealed that the difference was not significant. In addition, no proportional relation was observed between the concentrations of the tested drugs.

### 3.4. Effect of Ibuprofen and Diclofenac on Angiogenic Growth Factors’ Expression (VEGFA and HGF)

One of the essential components of bone repair is angiogenesis. Therefore, the mRNA expression level of vascular endothelial growth factor α (*VEGFA*) and hepatocyte growth factor (*HGF*) were measured by qRT-PCR in cells pre-treated with IBU and DIC for 24, 48, and 72 h. Data are presented as median values with error bars of 25–75% percentiles of two experiments from different donors (n = 4) performed in triplicate (*p* * ≤ 0.05 compared to the CTRL group).

**Figure 4:**
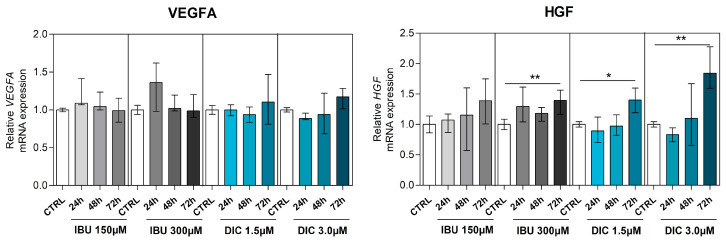
Expression of angiogenesis-associated genes—*VEGFA* and *HGF*. Effects of different concentrations of IBU and DIC on *VEGFA* and *HGF* mRNA expression in DPSCs at 24, 48, and 72 h. Target gene expressions are depicted relative to the control groups (DPSCs treated with equal solvents, ethanol in the medium for ibuprofen, or methanol in the medium for diclofenac). Data are presented as median values with error bars of 25–75% percentiles of two experiments from different donors (n = 4) performed in triplicate (*p* * ≤ 0.05 or *p* ** ≤ 0.01 compared compared to the CTRL group).

No statistically significant differences existed between untreated or treated DPSCs in *VEGFA* expression. Analysis of *HGF* expression revealed significant changes after pre-treatment with NSAIDs. Specifically, the HGF transcript was significantly upregulated in DPSCs cultured with IBU 300 µM (*p* ≤ 0.0040), DIC 1.5 µM (*p* ≤ 0.0392), and DIC 3 µM (*p* ≤ 0.0040) for 72 h (Figure 4).

### 3.5. Ibuprofen and Diclofenac Significantly Affect the mRNA Expression of Selected Genes in Apoptosis Signalling Pathways

To determine the effect of NSAIDs on genes associated with apoptosis, B-cell lymphoma 2 (*BCL2*), BCL2 associated X (*BAX*), BCL2 antagonist (*BAK*), caspase 3 (*CASP3*), and caspase 9 (*CASP9*) were analysed in DPSCs.

**Figure 5:**
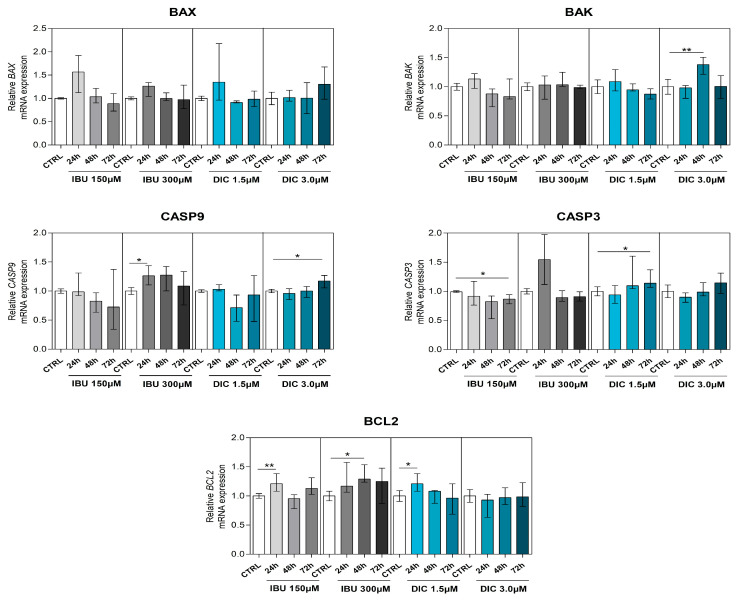
Expression of apoptosis-associated genes. Effects of different concentrations of IBU and DIC on *BAX*, *BAK*, *CASP9*, *CASP3*, and *BCL2* mRNA expression in DPSCs at 24, 48, and 72 h. Target gene expressions are depicted relative to the control groups (DPSCs treated with equal solvents, ethanol in the medium for ibuprofen, or methanol in the medium for diclofenac). Data are presented as median values with error bars of 25–75% percentiles of two experiments from different donors (n = 4) performed in triplicate (*p* * ≤ 0.05 or *p* ** ≤ 0.01 compared compared to the CTRL group), with marker expression as demonstrated by flow cytometry (n = 4).

*BAX* and *BAK*, members of the Bcl-2 family and regulators of the intrinsic apoptosis pathway, were slightly changed compared to CTRL. While there was no significant difference in *BAX* gene expression after pre-treatment, a significant upregulation of *BAK* expression was shown in DPSCs treated with DIC 3 µM for 48 h (*p* ≤ 0.0040) (Figure 5).

*CASP9* expression was significantly upregulated in DPSCs with the IBU 300 µM for 24 h (*p* ≤ 0.0162) and DIC 3.0 µM for 72 h treatments (*p* ≤ 0.0172). By evaluating the expression of *CASP3* in DPSCs, we found that IBU and DIC treatment led to diverse effects. While IBU 150 µM led to a significant decrease in *CASP3* expression in 72 h intervals compared to the CTRL (*p* ≤ 0.0121), IBU 300 µM had no significant effect. In contrast, DIC 1.5 µM significantly upregulated *CASP3* expression in 72 h (*p* ≤ 0.0162), whereas the DIC 3 µM treatment had no effect.

*BCL2*, a gene that prevents apoptosis and reduces oxidative stress, was significantly upregulated in DPSCs in almost all treatment regimens. IBU 150 µM led to a significant increase in *BCL2* expressions in 24 h intervals (*p* ≤ 0.0081) and IBU 300 µM in 48 h intervals (*p* ≤ 0.0107) compared to the control. DPSCs treated with DIC 1.5 µM had significantly increased *BCL2* expression in 24 h time intervals (*p* ≤ 0.0283) compared to the control group (Figure 5). Only the considerably changed expressions of genes are shown in Table 2.

## 4. Discussion

Bone healing after bone augmentation is a complex and coordinated process involving many different cell types and multiple cascades of mechanisms and signalling pathways [28,29]. Over the past several years, stem cell-based regeneration strategies have shown great promise for bone healing through endogenous restoration or exogenous transplantation of stem cells [30,31,32]. However, the therapeutic efficacy of stem cell-mediated regeneration is under solid control of the recipient microenvironment, which regulates resident MSCs and the regenerative efficacy of transplanted MSCs [33,34]. Therefore, there is a great effort to improve the endogenous microenvironment or to enhance exogenous MSC resistance, thus benefiting from transplanted MSCs. Besides those mentioned, bone healing can be affected by various other factors, such as the extent of damage, age or nutrition, and the administration of several pharmacological agents and an analgesic treatment [35].

Postsurgical pain is mainly driven by inflammation, with the production of prostaglandins in the periphery and central nervous system, which are the principal components of the initiation and propagation of pain [15]. Nonsteroidal anti-inflammatory drugs (NSAIDs), due to their anti-inflammatory, analgesic and antipyretic properties, are widely used in the treatment of postoperative pain [36,37]. Dental postsurgical pain is also mainly caused by inflammation, with cyclooxygenase-derived prostaglandins (PGs) being the significant sensitisers of free nerve endings, compared to other mediators of pain such as histamine, bradykinin, adenosine triphosphate, or low pH [38]. NSAIDs exert anti-inflammatory properties by inhibiting the synthesis of cyclooxygenase (COX) enzymes with a reduction in PGs’ production [39]. These lipid mediators play an important role in bone repair.

Moreover, they influence the regulation of inflammation, increase osteoblast proliferation and differentiation, and enhance osteoclast activity and bone resorption [18]. Accumulating evidence suggests that the general therapeutic effects of MSCs in bone repair are due to their abilities to promote a regenerative microenvironment and not because of their capabilities to differentiate and incorporate into the host tissue. The beneficial effects of MSCs involve immunomodulatory effects, stimulation of angiogenesis and antiapoptotic effects, and recruitment of host stem/progenitor cells into the site of bone repair [10,11]. However, to date, little is known about the effect of NSAIDs on the therapeutic potential of mesenchymal stem cells used in stem cell-based tissue engineering approaches. Therefore, the main research objective of the present study was to evaluate the in vitro effects of therapeutic doses of non-selective NSAIDs, ibuprofen and diclofenac, on the properties of human mesenchymal stromal cells isolated from dental pulp (DPSCs). Ibuprofen and diclofenac were chosen because of their popularity and long history of use as anti-inflammatory drugs and pain relievers in patients.

By previous studies [40,41,42,43], our analysis revealed that isolated cells from dental pulp were positive for the mesenchymal stem cell markers CD73, CD90, and CD105 and did not express markers typical for hematopoietic and endothelial cells, CD14, CD20, CD34, and CD45. Ibuprofen or diclofenac pre-treatment did not cause any significant alterations in the expression of the mentioned markers during the therapeutic dosage range. Meanwhile, doses of ibuprofen used in the study of Salkin and Basaran et al. (2022) were several times higher than therapeutic plasma concentration. In their research, high-dose ibuprofen (3 mmol/L) significantly increased CD73 expression in DPSCs [25], which indicates dosage-dependent results.

Regarding cellular viability, there is conflicting evidence in the literature regarding NSAIDs’ effect on stem cell viability. While doses of ibuprofen used in the study by Salkin and Basaran et al. (2022) were 0.1 mmol/L and 3 mmol/L, DPSCs’ viability increased significantly in the ibuprofen-applied groups [25]. However, different results were revealed with therapeutic concentrations of ibuprofen pre-treatment (25 µg/mL and 50 µg/mL) on human bone marrow mesenchymal stem cells. In the study of Kulesza et al. (2022), the authors demonstrated that higher ibuprofen doses negatively affected MSCs’ viability. However, the maximum decrease in cell viability was observed after ten days of treatment for 50 µg/mL of ibuprofen (92% relative viability) [17]. The conclusion of the study by Müller et al. (2011) demonstrated that the effects of NSAIDs on MSCs depend mainly on the concentrations used [44]. We did not prepare the dose-dependent research because the ibuprofen and diclofenac concentrations chosen in our study did not differ from the maximum serum concentrations during standard pharmacotherapy. Still, we found that NSAIDs have unfavourable effects on DPSCs’ viability. An ibuprofen concentration of 150 µM significantly suppressed the viability of DPSCs in the 72 h interval.

Similarly, diclofenac in doses of 3 µM significantly decreased cell viability in the 72 h interval of DPSCs’ pre-treatment. Comparable results were observed in the study of Kudo et al. (2003), who demonstrated that diclofenac (10 µM) induced the death of neural stem cells after 24 h treatment [45]. These differences between studies may result from the NSAIDs’ concentrations, different pre-treatment times, and the cell type and species used.

Over the past several years, cell-based therapies for bone regeneration have been extensively investigated [34]. A growing body of literature suggests that mesenchymal stromal cells may secrete factors that support angiogenesis at the site of injury, which presents an essential component of bone repair [46,47]. Angiogenesis is regulated by various growth factors, hormones, cytokines, and low-molecular-mass mediators [48]. A significant role is played by vascular endothelial growth factor α (*VEGFA*), produced mainly by inflammatory cells and stromal cells to induce blood vessel growth. The various cellular functions of VEGF result from its ability to initiate a diverse, complex network of signalling pathways [49]. Indeed, in bone healing, VEGF increases endothelial cell differentiation and proliferation, tube formation, and the mobilisation and recruitment of endothelial progenitor cells [47]. Paracrine VEGF signalling is mediated by the tyrosine kinase receptors VEGFR1 and VEGFR2. All VEGF isoforms can bind to both receptors. However, VEGFR2 has strong tyrosine kinase activity and thus is the main receptor involved in cell signalling, including the activation of mitogen-activated protein kinase (MAPK), phosphatidylinositol 3-kinase (PI3K/Akt), and Src and Rac signalling [50]. Hepatocyte growth factor (HGF) is another critical pleiotropic cytokine involved in numerous complex biological processes in tissue regeneration, tumour growth, and angiogenesis. Binding and activating its c-met receptor, expressed in several cell types, triggers several signalling pathways, such as PI3K/Akt, MAPK, and others, most of which are expected to lead to VEGFA signalling [51]. Regarding angiogenesis, HGF stimulates endothelial cells directly through the c-Met receptor and indirectly by facilitating the expression of other angiogenic factors represented by VEGF [52].

To investigate the impact of ibuprofen and diclofenac on the secretory activity of DPSCs, we performed a qRT-PCR analysis of selected gene expressions. DPSCs are known to secrete numerous bioactive factors, and this activity is crucial for their immunomodulatory and pro-regenerative angiogenic abilities. First, we evaluated the effect of low- and high-dose ibuprofen and diclofenac on the mRNA expression level of *VEGFA*. The results indicated that neither ibuprofen nor diclofenac significantly changed the expression of the most important angiogenic factor, *VEGFA*. However, the analysis of the *HGF* transcript revealed the significant effect of NSAIDs on DPSCs. While 150µM ibuprofen did not affect *HGF* expression, a 300 µM dose of ibuprofen and both used diclofenac doses caused a significant increase in the *HGF* transcript level in DPSCs after the 72 h interval of pre-treatment. The effects of ibuprofen preconditioning have already been reported regarding MSCs isolated from bone marrow. Kulesza et al. (2022) evaluated the consequence of ibuprofen on MSCs’ secretome by using Proteome Profiler and Luminex immunoassays. Ibuprofen (25 µg/mL for 72 h) significantly decreased the mean secretion of VEGF (by 20%) and HGF (by 31%) compared to the secretion of control MSCs [17]. Similarly, reduced VEGFA expression was observed in osteoblast cells treated with 10 μM doses of diclofenac and ibuprofen at 24 h of treatment, evaluated by RT-PCR and compared with the expression of untreated osteoblasts [53]. These results indicate that NSAIDs modulate the expression of angiogenic factors.

Previous studies have shown that NSAIDs are implicated in the apoptosis and death of cells and tissues and can have anti-cancer effects. However, the mechanism of this effect is not well known in molecular and cellular terms [54]. The reduction in the viability of cancer cells as well as the activation of caspase pathways were confirmed in ibuprofen pre-treated cervical cancer cells [55]. A study by Akrami et al. (2014) evaluated the impact of ibuprofen on the expression of set genes involved in apoptosis. The results revealed that ibuprofen at 500 µM downregulated the transcription of the *BCL2* gene in gastric cancer cells [56]. Another study revealed that neural stem cells treated with diclofenac (60 µM) for 24 h showed nuclear condensation, and Western blot analysis reported that the activation of caspase 3 was increased by treatment with diclofenac in a concentration-dependent manner (10 µM, 30 µM, and 60 µM) [45].

Regarding the effects on genes involved in apoptosis, we found that NSAIDs used in our study alter the transcription of selected genes, except for *BAX*, where no changes after pre-treatment were observed. Firstly, we evaluated the effect of 150 µM ibuprofen, which significantly downregulated expression of caspase 3 in a 72 h interval, while 300 µM ibuprofen increased the transcription of caspase 9 after 24 h. Both used concentrations caused an upregulation of the *BCL2* gene in DPSCs in early intervals. BAK, CASP3, and CASP9 expressions were significantly increased in the diclofenac pre-treatment group compared to the untreated group. The diclofenac dose of 1.5 µM significantly increased *BCL2* expression in a 24 h interval. However, in the 3 µM diclofenac group, this effect was not reported. The difference between ibuprofen and diclofenac’s effects on the induction of cell death may be explained by the selectivity of COX-2 inhibition. Although diclofenac is generally accepted as a traditional NSAID in the published literature, it was proven to have a higher selectivity for COX-2 than for COX-1, in contrast with most traditional NSAIDs. The degree of COX-2 selectivity demonstrated for diclofenac can be compared to that of celecoxib, a selective COX-2 inhibitor [23,57,58]. Many experimental and clinical studies have suggested that COX-2 inhibitors may reduce cancer risk through the induction of apoptosis [54,59]. The changes in *HGF*, *BAK*, *CASP3*, *CASP9*, and *BCL2* expression, combined with the reduced viability of NSAID-treated DPSCs, indicate various effects on stem cell properties that might affect the therapeutic outcome. Unfavourable effects of both ibuprofen and diclofenac on viability and apoptosis-related genes in stem cells may help in designing better pharmacotherapy strategies and highlight the need for increased caution in the use of NSAIDs in postsurgical pain therapy after regenerative treatment with DPSCs.

## 5. Conclusions

The beneficial effects of MSCs in bone repair involve immunomodulatory effects, the stimulation of angiogenesis and antiapoptotic effects, and the recruitment of host stem/progenitor cells into the site of bone repair. Our results showed that ibuprofen and diclofenac at concentrations achieved in the serum of patients during standard pharmacotherapy do not significantly affect the expression of mesenchymal stem cell markers. However, we demonstrated that both ibuprofen and diclofenac significantly decrease the viability of DPSCs, yet these significant decreases were approximately 2.5%, and therefore additional studies are needed.

Moreover, our results revealed that used NSAIDs do have an impact on the gene expression of DPSCs. The dose of 150 µM of ibuprofen decreased *CASP3* levels after 72 h of treatment and increased *BCL2* after 24 h. The dose of 300 µM of ibuprofen increased levels of *HGF* after 72 h, *CASP3* after 24 h, and *BCL2* after 48 h of treatment. The doses 1.5 µM and 3 µM of diclofenac increased *HGF* transcript levels after 72 h; 1.5 µM of diclofenac treatment increased *CASP3* after 72 h and *BCL2* after 24 h. A dose of 3 µM of diclofenac increased *BAK* after 48 h and *CASP9* after 72 h.

These results suggest that the concomitant use of ibuprofen or diclofenac with stem cell-based tissue engineering approaches might impact the therapeutic outcome of the procedure by decreasing the viability and altering the expression of apoptosis-related genes in stem cells. Thus, this knowledge may help design better pharmacotherapy strategies and highlight the need for increased caution in their use after regenerative treatment with DPSCs. However, further, more extensive studies are required to verify these hypotheses.

## Figures and Tables

**Table 1:** The probes for the genes.

Vascular endothelial growth factor alpha (VEGFA; Hs00900055_m1)
hepatocyte growth factor (HGF; Hs00300159_m1)
B-cell lymphoma 2 associated X (BAX; Hs00180269_m1)
B-cell lymphoma 2 antagonist 1 (BAK; Hs00832876_g1)
caspase 9 (CASP9; Hs00962278_m1)
caspase 3 (CASP3; Hs00234387_m1)
B-cell lymphoma 2 (BCL2; Hs00608023_m1)
beta-2-microglobulin (B2M; Hs99999907)
glyceraldehyde-3-phosphate dehydrogenase (GAPDH; Hs99999905)

**Table 2:** Significant changes in gene expressions.

	*HGF*	*BAK*	*CASP9*		*CASP3*	*BCL2*	
	72 h	48 h	24 h	72 h	72 h	24 h	48 h
150 µM IBU					**↓***p* ≤ 0.0121	**↑***p* ≤ 0.0081	
300 µM IBU	**↑***p* ≤ 0.0040		**↑***p* ≤ 0.0162				**↑***p* ≤ 0.0107
1.5 µM DIC	**↑***p* ≤ 0.0392				**↑***p* ≤ 0.0162	**↑***p* ≤ 0.0283	
3 µM DIC	**↑***p* ≤ 0.0040	**↑***p* ≤ 0.0040		**↑***p* ≤ 0.0172			

**↑** up-regulated expression compared to the CTRL; **↓** down-regulated expression compared to the CTRL.

## Data Availability

The data that support the findings of this study are available upon request.

## Abbreviations

NSAIDs	nonsteroidal anti-inflammatory drugs
DPSCs	dental pulp stem cells
HGF	hepatocyte growth factor
VEGFA	vascular endothelial growth factor alpha
CASP9	caspase 9
BCL2	B-cell lymphoma 2
CASP3	caspase 3
BAK	B-cell lymphoma 2 antagonists 1
BAX	B-cell lymphoma 2 associated X
IBU	ibuprofen
DIC	diclofenac
FBS	foetal bovine serum
DMEM	Dulbecco’s modified Eagle medium
ISCT	International Society for Cellular Therapy
PI	propidium iodide
MTT	tetrazolium salt
